# Sleep Deprivation and Alzheimer’s Disease: A Review of the Bidirectional Interactions and Therapeutic Potential of Omega-3

**DOI:** 10.3390/brainsci15060641

**Published:** 2025-06-14

**Authors:** Nasar Ullah Khan Niazi, Jiahui Jiang, Haiyan Ou, Ruiye Chen, Zhiyou Yang

**Affiliations:** 1 College of Food Science and Technology, Shenzhen Institute of Guangdong Ocean University, Guangdong Provincial Key Laboratory of Aquatic Product Processing and Safety, Guangdong Ocean University, Zhanjiang 524088, China; nasark@stu.gdou.edu.cn (N.U.K.N.); 2112203057@stu.gdou.edu.cn (J.J.); 2112203092@stu.gdou.edu.cn (H.O.); 2112203075@stu.gdou.edu.cn (R.C.); 2 Zhanjiang Municipal Key Laboratory of Marine Drugs and Nutrition for Brain Health, Institute of Marine Drugs and Nutrition, Guangdong Ocean University, Zhanjiang 524088, China

**Keywords:** sleep disorders, Alzheimer’s disease, comorbidity, Omega-3 fatty acids, amyloid processing

## Abstract

Sleep is essential for physical and mental health, playing a critical role in memory consolidation, behavioral stability, and the regulation of immune and metabolic functions. The incidence of sleep disorders, particularly sleep deprivation (SD), increases with age and is prevalent in neurodegenerative and psychiatric disorders such as Alzheimer’s disease (AD). Nearly 40% of AD patients experience significant chronic sleep impairments. The clinical distinction between late-life sleep disorders and AD is often challenging due to overlapping symptoms, including cognitive decline and behavioral impairments. Although the exact causal relationship between SD and AD remains complex and multifaceted, strong evidence suggests a bidirectional link, with AD patients frequently exhibiting disrupted sleep architecture, reduced slow-wave activity, and shorter total sleep duration. On a pathophysiological level, SD contributes to neuroinflammation, amyloid-β plaque deposition, and tau tangles, which are key features of AD. Current treatments, such as sedatives and antidepressants, often have limitations, including inconsistent efficacy, dependency risks, and poor long-term outcomes/recurrence, highlighting the need for safer and more effective alternatives. This review examines the interplay between SD and AD and proposes omega (n)-3 fatty acids (FAs) as a potential therapeutic intervention. Preclinical and clinical studies suggest that n-3 supplementation may improve sleep onset/quality, reduce neuroinflammation, support synaptic function, and decrease amyloid-β aggregation, thereby alleviating early AD-related neurological changes. Given their safety profile and neuroprotective effects, n-3 FAs represent a promising strategy for managing the comorbidity of sleep disorders in AD.

## 1. Introduction

Sleep and aging are two inevitable parts of life. Physiological aging is commonly accompanied by alterations in sleep architecture, characterized by increased sleep fragmentation, frequent nocturnal arousals, and a greater predisposition to daytime sleepiness [1]. Sleep disruption is a big problem not only for patients but also for their caregivers, which further increases the associated risks among the demography [2]. Interestingly, age-related anatomical and neurochemical changes in the brain are also associated with dementia [3]. On the basis of age, dementia can be categorized as early-age dementia and late-age dementia. Frontotemporal dementia (FTD) is considered the main cause of early-age dementia, as 50% of dementia patients who are under the age of 60 suffer from FTD [4,5]. Alzheimer’s disease (AD) is the most prevalent cause of old-age dementia, and 60–80% of elderly dementia patients suffer from AD [6]. AD is a biological or clinical–biological construct characterized by the presence of core AD biomarkers (i.e., Aβ40/42 and pTau) and specific clinical phenotypes, such as posterior cortical atrophy, logopenic aphasia, hippocampal type amnestic syndrome, and objective cognitive/behavioral deficits [7]. Due to changing lifestyles and the increasing socioeconomic burden, as well as aging populations around the globe, there is a gradual but consistent increase in patients suffering from sleep deprivation (SD) and AD. Overall, 45–90% of dementia patients suffer from insomnia or sleep disruptions [8], whereas 21–45% of AD patients suffer from sleep disruptions [8,9,10]. This represents a strong correlation between cognitive decline and sleep disorders, which usually manifest in the early stages of AD. Thus, it is important to decipher the possible links between AD and sleep; therefore, the current review will focus on SD-induced neuroimmunological and molecular aspects and AD.

Sleep disorders in AD are complex and bidirectional. AD employs different factors, such as dysregulation of the circadian rhythm [11], degeneration of neural circuits/regions important for the regulation of the sleep–wake cycle [12], and changes in sleep architecture [13]. As a consequence, sleep disruptions exacerbate memory deficits and impair memory consolidation by facilitating neuroinflammation and altering glial polarizations to the proinflammatory state, reducing amyloid-β (Aβ) clearance, increasing tau hyperphosphorylation, and accelerating the neuronal apoptosis; all of which lead to neurodegeneration and ultimately AD.

**Figure 1:**
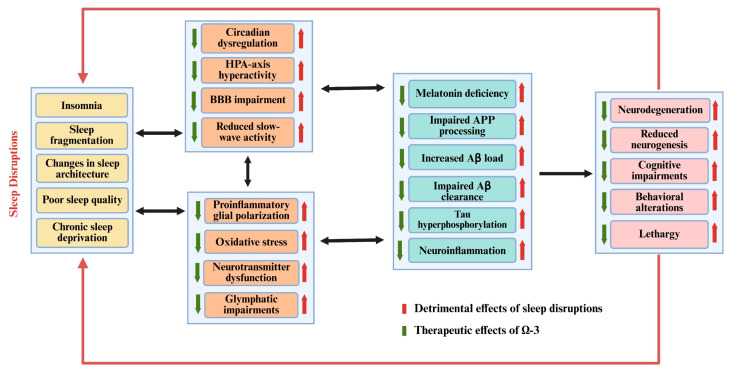
Schematic diagram of bidirectional relationship between sleep disorders, AD, and therapeutic potential of n-3 FAs.

Although there are many treatments available to address sleep disruptions and AD, they come with their own set of limitations, including addiction, recurrence, inefficiency, and individual variations in therapeutic response. To address these limitations, omega (n)-3 fatty acids (FAs) can be used as a safe and effective therapeutic alternative, as studies have reported that n-3 can ameliorate the aforementioned neuropathological changes associated with SD and early-stage AD. This review will help to elucidate the sleep-related changes in normal aging and AD and the pathological similarities between SD and AD, especially with reference to neuroinflammatory changes, amyloid processing, and the therapeutic application of n-3 FAs due to their wide range of biological activities for treating SD and AD. Finally, recommendations for future studies will be shared. A brief schematic diagram of the reported interactions is shown below (Figure 1):

## 2. Sleep-Related Cognitive Changes in Healthy Aging

**Figure 2:**
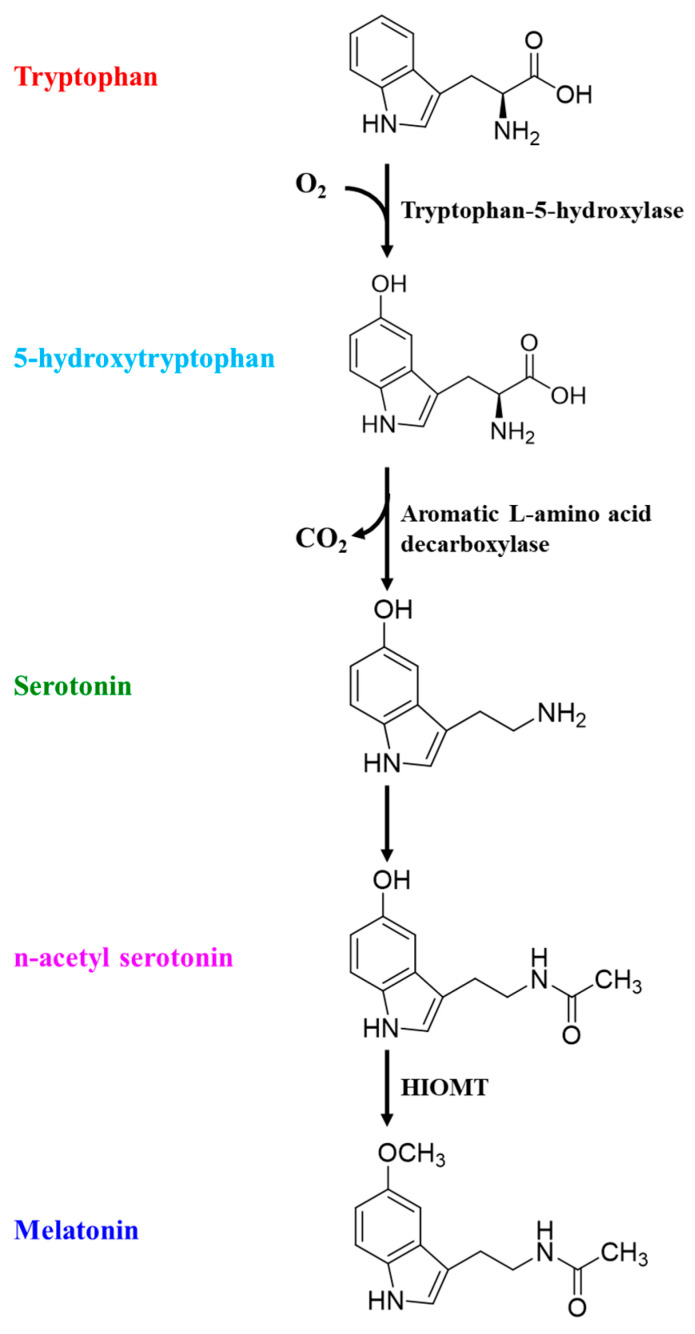
Physiological conversion of tryptophan into melatonin.

The incidence of sleep disruptions increases with age as the sleep quality gets worse due to well-documented changes in sleep patterns and sleep architecture [14,15]. Poor sleep among the elderly is characterized by delayed onset, decreased latency, difficulty in maintaining sleep, nocturnal shifts in sleep patterns, and overall decreased sleep duration. Polysomnographic studies indicate that aging shifts the pattern and duration of sleep stages as the length of non-rapid eye movement (NREM) sleep stages 1 and 2 (light sleep) increases. Although rapid eye movement (REM) sleep changes also happen, these changes occur later in old age [16]. The age-related micro-architectural changes in sleep structure include decreased sleep spindles and lower k-complex peaks [17]. Collectively, these changes are attributed to increased lethargy and daytime sleepiness. Age-related neurochemical changes in the brain lipid profile, especially decreased brain n-3 contents [18], not only exert a neuroinflammatory pressure but also decrease the bioavailability of tryptophan, which is a precursor to 5-hydroxytryptophan (5-HT, serotonin) [19]. Aging-induced neuroinflammation can also suppress 5-HT production, as indoleamine 2,3-dioxygenase (IDO) activity is upregulated by inflammatory microglia and proinflammatory cytokines [20]. Serotonin is later converted into melatonin, which plays an important role in sleep induction and sleep duration, as studies have pointed towards decreased melatonin concentrations in old age [21] (Figure 2).

Studies have also indicated a strong link between behavioral/memory impairments and sleep alterations [22], and polysomnography can aid in distinguishing between different conditions. AD patients typically exhibit reduced delta slow-wave activity (SWA) during NREM sleep, particularly during REM sleep [23,24,25]. These polysomnographic distinctions, while not absolute, can provide supportive data when combined with other clinical diagnostic tools used for AD diagnosis.

The incidence of neuroinflammation increases with age and is highly correlated with memory impairments and sleep disruptions. Glial cells, especially microglia and astrocytes, play an important role in age-related neuroinflammatory changes and are likely involved in the interactions between sleep quality and neuroimmunological changes. In response to SD, microglia exhibit a de-ramified morphology [26] indicative of their activation and polarize into an inflammatory state [27]. However, it is a bidirectional relationship, as research indicates the role of microglia in sleep onset and depth as well. According to studies, SWA diminishes after the administration of the microglial inhibitor minocycline [28]. In another study, intraperitoneal administration of minocycline resulted in a rapid enhancement of wakefulness and a marked reduction in NREM sleep. Moreover, minocycline suppresses the enhancement of NREM-sleep delta power, which serves as an alternative marker to measure sleep depth [29].

Inflammatory microglial phenotype M1 is distinguished by the expression of CD11b, CD68, and proinflammatory cytokines, including tumor necrosis factor (TNF)-α, interleukin (IL)-6, and IL-1β. These cytokines have been shown to induce oxidative stress, hypothalamic–pituitary–adrenal (HPA) axis hyperactivity, neuronal damage, neurotransmitter dysfunction, and, in the end, memory impairments. Notably, increased plasma TNF-α concentrations have also been reported in sleep-deprived mice [30,31]. Conversely, TNF inhibitor administration prior to sleep results in diminished sleep rebound in animals compared to controls, leading to sleep disturbances [31], which highlights the delicate homeostatic balance between sleep and proinflammatory cytokines. The anti-inflammatory microglial phenotype M2 may enhance the expression of anti-inflammatory cytokines, including IL-10, IL-4, transforming growth factor-β1, and arginase-1, thereby mitigating inflammation and safeguarding neurons. Our previous study reported that the activation of M2 microglia was reduced in chronic sleep deprivation (cSD) [27], which indicates that cSD likely reduces the homeostatic neuroprotective efficacy of the brain. With regard to other glial cells, the astrocytes M1 and M2 modulate the A1 and A2 phenotypes, respectively. Astrocytes offer trophic support to neurons and facilitate synapse formation and function, an important aspect of AD pathogenesis [32]. Astrocytes rapidly respond to inflammation, express receptors for immunomodulators, and synthesize substances that regulate sleep, such as adenosine and prostaglandins, in response to immune challenges [33]. Altered gliotransmission of astrocytes leads to diminished sleep pressure, suggesting astrocyte-derived adenosine, an adenosine triphosphate metabolite, as a potential molecular basis for this phenomenon [34]. Emerging evidence from SD-related studies reveals varying outcomes depending on the experimental protocols and SD duration. While acute and sub-chronic SD predominantly enhances M2/A2 astrocyte polarization activation [27] and demonstrates neurotrophic potential through elevated concentrations of glial cell line-derived neurotrophic factor and brain-derived neurotrophic factor (BDNF) [35], cSD paradoxically reverses these neuroadaptive responses [36]. Notably, the observed downregulation of these critical neurotrophic factors mirrors the pathophysiological characteristics of neurodegenerative disorders, particularly AD, suggesting a potential mechanistic link between chronic sleep disruption and neurodegeneration.

## 3. Pathogenesis and Hypotheses of AD

While AD’s etiology remains unclear, several hypotheses have emerged to explain the possible pathogenicity of this disease. Those most influenced by sleep disruptions are discussed below.

### 3.1. Neuroinflammatory Hypothesis of AD

**Figure 3:**
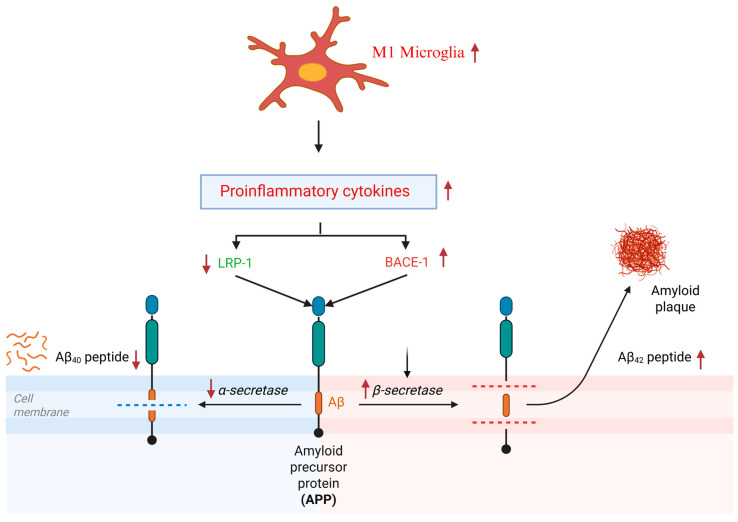
Neuroinflammation and homeostatic processing of APP. ↑ means upregulated, ↓ means downregulated.

Earlier epidemiological studies indicated that non-steroidal anti-inflammatory drugs reduced the chances of developing AD [37,38]. Subsequently, many in vivo and clinical studies were conducted to elucidate these findings, but significant inconsistencies in the results were found [39,40]. Nonetheless, these studies helped to establish the foundational link between neuroinflammation and AD. Subsequent research confirmed that neuroinflammation drives key AD pathologies, including memory deficits, Aβ deposition, tau tangles, neuronal apoptosis, and neurodegeneration [41,42,43]. From a neuroimmunological standpoint, glial cells—particularly microglia and astrocytes—serve as the primary immune regulators in the brain, while oligodendrocytes primarily function in neuronal myelination [44,45]. Sustained M1 activation leads to the release of proinflammatory cytokines and oxidants, including reactive oxygen species (ROS) and nitric oxide (NO). Notably, a triggering receptor expressed on myeloid cell type 2 (TREM2), a microglial marker, exhibits mutations in AD patients [46]. The TREM2 R47H variant was identified in late-onset AD, conferring a 2–4 fold increased risk, and represents the strongest genetic association with AD after apolipoprotein E (ApoE)-4 [47]. In response to a neuroinflammatory stimulus, microglia transition from a resting to a reactive state. While acute microglial activation promotes Aβ clearance, chronic activation exacerbates AD pathology by enhancing the release of pro-neuroinflammatory mediators [48]. This impairs phagocytic efficiency by reducing glial-dependent Aβ binding and clearance capacity, accelerates plaque deposition, and [49] disrupts the homeostatic processing of amyloid precursor protein (APP). Studies have reported that the low-density lipoprotein receptor-related protein (LRP)-1 and beta-site amyloid precursor protein cleaving enzyme (BACE)-1 are key players in AD pathogenesis as they facilitate the production, deposition, and clearance of Aβ in the brain. LRP1 and BACE1 compete for APP metabolism and produce APPα and APPβ, which later facilitate the production of Aβ_40_ and Aβ_42_, respectively [50] (Figure 2). M1 microglial polarization can reduce LRP-1 production, which can subsequently increase BACE-1 activation in neuroblastoma cells [51]. Importantly, our previous study indicated that cSD-induced neuroinflammation decreased the APPα/APPβ ratio in the mouse hippocampus, which is important for monitoring the overall Aβ status and can be further developed as a possible diagnostic tool for non-invasive diagnosis of AD in humans [27,52] (Figure 3). The receptor for advanced glycation end products (RAGE), another key regulator of BACE-1, not only perturbs Aβ in cerebral vessels, neurons, and microglia but also upregulates BACE-1 activity via the nuclear factor of activated T-cells-1. It activates nuclear factor kappa B (NFκB), which later enhances the release of proinflammatory cytokines, including RAGE itself, creating a positive feedback loop [53,54]. Studies suggest that elevated RAGE is also positively correlated with sleep disruptions in humans [55].

IL-1, released by brain-resident microglia, acts as a neuroinflammatory master regulator by inducing downstream inflammatory cytokines (e.g., IL-6 and TNF-α) [56]. Elevated concentrations of IL-1α, one of the two principal IL-1 subtypes, have been associated with up to a three-fold elevation in AD risk [57]. Whereas the deficiency of IL-1β, the other major IL-1 subtype, has been shown to significantly delay the initiation of neuroinflammatory processes and the progression of neurodegenerative pathology [58]. IL-1β binds to IL receptor type 1, which triggers the recruitment of the IL-1-receptor accessory protein from the active signaling complex [59], expressed widely across neuronal and glial populations with particularly dense localization in the hippocampal pyramidal tract and dentate gyrus. These regions are critically involved in learning and memory processes and are among the earliest affected regions in AD pathogenesis [60]. Furthermore, IL-1β upregulates APP synthesis in glial cells and enhances APP processing via protein kinase C activation [61,62]. IL-1β participates in a self-perpetuating cycle where elevated IL-1β levels enhance Aβ load and plaque deposition, subsequently activating microglia and promoting further IL-1β production [63]. Moreover, in the brain and plasma of AD patients, elevated TNF-α levels have also been reported. TNF-α binds to two receptors: tumor necrosis factor receptor 1 (TNFR1) and TNFR2. Studies indicate that increased expression of TNFR1, induced by NFκB and Aβ, in the brain is essential for neuronal apoptosis/neurodegeneration [64]. Conversely, reduced TNFR1 levels correlate with decreased plaque deposition and microglial activation in the hippocampus, leading to improved cognition [65]. Moreover, in the cerebrospinal fluid (CSF) of AD patients who were reported to suffer from mild cognitive impairment (MCI) 6 years prior to AD, elevated concentrations of TNFR1 and TNFR2 were also observed [66]. In conjunction, neuroinflammatory changes are of critical importance in the pathogenesis of AD.

### 3.2. Aβ Hypothesis of AD

As discussed earlier, Aβ deposition and plaque formation are key features of AD and, together with other disease markers, have driven the dominant hypothesis since the 1990s. Central to this hypothesis are Aβ plaques and the overexpression of APP, both of which are well-documented in postmortem studies of AD patient’s brains. Aβ peptides are the main component of Aβ plaques and consist of 36–43 amino acids. As mentioned above, the Aβ is a byproduct of APP catabolism by the activity of α, β, and γ secretase in the amyloidogenic pathway [67]. Since the development of the first transgenic mice to analyze the APP metabolism and Aβ plaque formation in 1995, many studies have been conducted that further validate this hypothesis. While Aβ_40_ is produced due to LRP-1 activity, as shown above, in normal physiological conditions, Aβ_42_ is produced from APP via the sequential proteolytic cleavage by β-secretase followed by γ-secretase. BACE1 cleaves APP to produce a C-terminal fragment, which γ-secretase then cleaves to generate Aβ peptides [68]. In response to APP catabolism under normal circumstances, Aβ_40_ makes up to 90% of the total Aβ peptides, while the remaining 10% of Aβ peptides are Aβ_42_ [69]_._ Although both of these peptides are found in AD, compared to Aβ_40_, which can be easily removed from the brain, Aβ_42_ is known for its hydrophobic and neurotoxic properties, and it aggregates and forms Aβ plaques in the brain, causing neurodegeneration [70]. Neurons, especially in the cornu-ammonis-1 region of the hippocampus and other AD-prone regions, accumulate Aβ preceding extracellular Aβ plaque formation and neurofibrillary tangles (NFTs) [71,72]. Aβ oligomers lead to cognitive impairment by inhibiting the long-term potential of hippocampal tissue and synaptic plasticity [73]. Previous studies have reported that the administration of Aβ significantly reduced SH-SY5Y cell viability, nerve growth factor (NGF), tyrosine kinase (Trk)-A receptor expression, and antioxidant glutathione (GSH) activity while increasing ROS, NO, and TNF-α and reducing BDNF/Trk-B [74,75]. Aβ also induced neuronal apoptosis, as indicated by the dysregulation of the Bax/Bcl-2 ratio and increased caspase-3 expression, all of which are pathological hallmarks of AD [76,77]. In sum, these studies add to the strengths of the Aβ hypothesis.

### 3.3. Tau Hyperphosphorylation in AD

Tau is a soluble microtubule-binding protein and has strong binding potential with phosphate groups. This protein is abundantly present in the neuronal axons and helps to maintain the structural integrity and shape of microtubules. It also promotes nutrient and other cell-to-cell transportation across the cell membrane [78]. In AD brains, excessive hyperphosphorylation of tau is reported, which converts normal tau to NFTs and paired helical filament (PHF)-tau. Moreover, all six tau isoforms (0N3R, 1N3R, 2N3R, 0N4R, 1N4R, and 2N4R) have been reported in the formation of NFTs and PHF-Tau. In normal healthy subjects, phosphorylated (p)Tau concentrations increase with aging [79]. Several factors associated with sporadic AD, such as head trauma, psychological and physiological stress, and insulin resistance, contribute to the disruptions in calcium regulation and glycogen synthase kinase (GSK) 3β signaling. Dysregulation of calcium homeostasis initiates tau phosphorylation at early stages and predisposes tau to subsequent hyperphosphorylation. This priming effect is mediated by the inhibition of GSK3β at critical diagnostic sites. Consequently, this process facilitates the production of tau fibrils, which are the primary structural components of NFTs. The evidence also suggests that pTau can propagate between neurons within cortical networks that support cognition [80].

**Figure 4:**
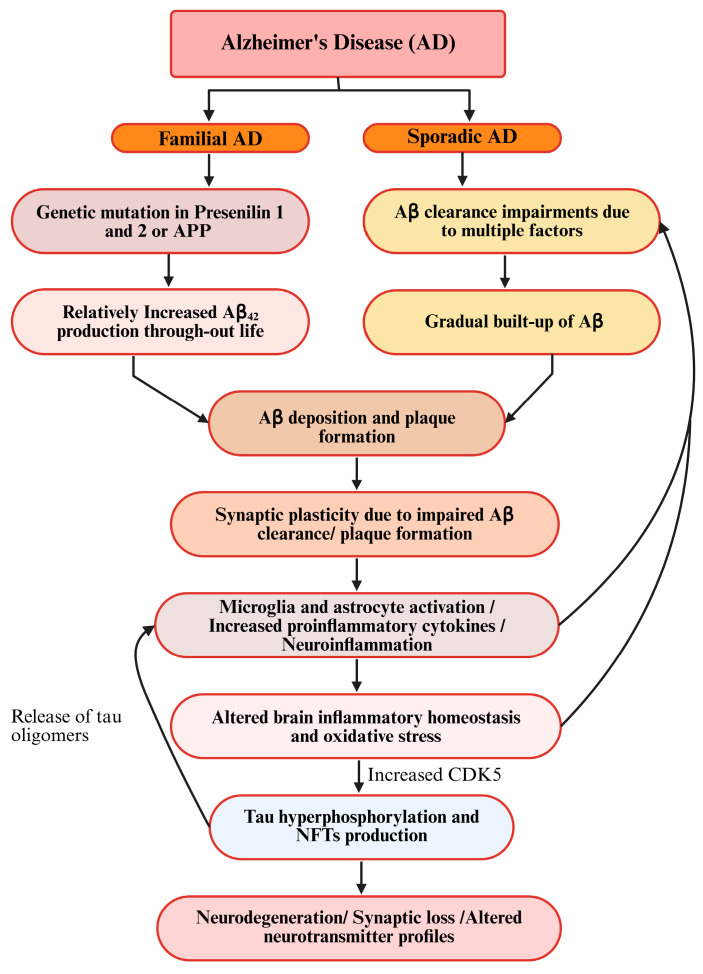
Schematic diagram of prevailing hypotheses for AD.

A neuroinflammation-induced increase in proinflammatory cytokines can also initiate or facilitate tau hyperphosphorylation. Studies suggest that IL-6 and IL-1β can activate cyclin-dependent kinase-5 (CDK-5), which is known for tau hyperphosphorylation. Regarding other AD hallmarks, pTau and amyloid-beta (Aβ) exhibit a reciprocal relationship [81]. Aβ initiates tau’s transformation into a toxic form in AD, but toxic tau also amplifies Aβ deposition through a feedback loop. Later on, both Aβ and tau can self-propagate and spread across the brain via prion-like mechanisms, driving neurodegeneration [82,83] (Figure 4).

Recent studies have highlighted the clinical importance of tau as a possible early biomarker of AD. Reports show that pTau217 and pTau181 can be detected in blood as a noninvasive method for authentic early diagnosis of AD [84,85]. Both have been approved by the FDA as an aid in the diagnostic evaluation of AD. A blood pTau217 immunoassay showed comparable accuracies to CSF biomarkers in identifying tau and Aβ pathologies. Longitudinally, plasma p-tau217 values were annually increased in both Aβ- and tau-positive individuals, even at the preclinical stage of AD [84]. The correlation between Aβ and tau pathology is complex and not fully understood, and tau pathology generally correlates better with a clinical course [86]. A systematic review and meta-analysis including 2764 Aβ-positive (A+) and 5646 Aβ-negative (A−) subjects showed higher mean blood p-tau181 values in the A+ group than in the A− group [87]. These findings highlight that pTau is an effective biomarker for early-onset AD.

### 3.4. Other Hypotheses for AD

Many other hypotheses and theories have been presented as possible etiologies of AD. Acetylcholine (ACh) deficiency is well-documented and is a target for current AD treatments [88,89]. Additionally, oxidative stress and mitochondrial dysfunction are pivotal contributors to apoptotic changes in the brain, leading to neurodegeneration [90]. Moreover, new evidence has highlighted a positive correlation between dysbiosis of oral microbiota and AD [91,92].

Thus, therapeutic treatment that can effectively ameliorate neuroinflammation, Aβ plaque deposition, and tau hyperphosphorylation is of key importance.

## 4. The Neuropathological Comorbidity Between SD and AD

Sleep quality and circadian rhythmicity progressively decline in parallel with cognitive deterioration and the progression of AD pathology [93]. Current interventions appear to improve sleep architecture, enhance circadian regulation, and produce measurable but limited improvements in neuropsychological test performance, particularly in cognitive domains typically impaired in AD [94,95]. While these findings suggest potential benefits for sleep-disturbed cognitively impaired populations, the therapeutic efficacy appears constrained to the early disease stages [96].

Emerging evidence suggests a bidirectional relationship between sleep disturbances and AD pathogenesis, as poor sleep accelerates amyloid deposition, and amyloid pathology disrupts sleep regulation [42]. While a clinical study established that poor sleep quality correlates with higher Aβ plaque deposition even in cognitively normal individuals [97], experimental research reveals that even acute sleep deprivation (SD) significantly increases CSF Aβ42 levels in healthy mice [98]. Notably, once Aβ plaques form, they disrupt key regulatory mechanisms of both sleep–wake cycles and circadian rhythms across species, as demonstrated in both murine models and human clinical studies. This creates a pathogenic feedback loop where (1) sleep impairment accelerates Aβ accumulation, and (2) established Aβ pathology further degrades sleep quality [99]. SWA, a characteristic electrophysiological feature of deep NREM sleep, represents a key mechanism for Aβ regulation. Electroencephalographic analyses in humans demonstrate that SWA reflects periods of reduced synaptic firing [49], which is particularly relevant as neuronal activity drives the release of soluble Aβ into the interstitial space. This neurophysiological relationship suggests that SWA may facilitate Aβ clearance during quiescent synaptic periods, potentially through enhanced glymphatic system function during deep sleep stages [100]. Actigraphy data from a study showed that sleep fragmentation significantly increases AD risk. Greater sleep disruption correlated with higher AD incidence, independent of age and ApoE-4 status, suggesting sleep quality may be an independent AD risk factor [101].

Amyloid plaques typically accumulate 10–15 years before observable cognitive impairment. AD transgenic mouse models (APP/PS1) demonstrate a compelling temporal association between sleep disruption and amyloid pathology [102]. These studies revealed that hippocampal and cortical Aβ plaque deposition coincides with increased wakefulness and sleep fragmentation and correlates with progressive sleep–wake cycle deterioration. Another study found SD increased CSF tau by >50%, followed by amyloid elevation [103]. In conjunction, these studies strongly suggest a feed-forward relationship between sleep impairments and AD progression [104].

These aforementioned studies demonstrate an SD-AD association, but amyloid plaques alone show an imperfect clinical correlation with AD. Combined with tau analysis, it can provide a better clinical diagnosis of cognitive impairment [105]. While the current evidence links SD to enhanced AD biomarker expressions, whether SD directly causes AD remains unproven and is under active investigation. An important fact is that current sleep–AD research predominantly employs rodent models. Mice exhibit nocturnal polyphasic sleep (brief, frequent bouts), contrasting with human monophasic patterns [106]. Crucially, core neurochemical mechanisms regulating sleep–wake cycles—including glutamatergic, GABAergic, and monoaminergic pathways—remain highly conserved across mammals, validating translational relevance for fundamental sleep–AD interactions. The hβAPP transgenic mouse model recapitulates the key aspects of AD progression, demonstrating the age-dependent deterioration of both circadian rhythms and sleep architecture that mirrors the temporal progression of human AD pathology [102].

With reference to SD as an inducer of AD-like cognitive decline, the combination of defensive activation theory (DAT) and active inference theory (AIT) provides a plausible explanation. DAT proposes defensive activation to maintain the anatomical and functional integrity of brain regions, and the role of sleep in maintaining this mainly covers the NREM- and REM-related defense matrix for memory preservation [107,108], whereas AIT highlights the intricate relationship between sensory inputs and perception, and these sensory inputs and perception coding in the brain are impaired due to neurodegenerative conditions such as AD [109]. In conjunction, both these theories can provide plausible explanations as understanding the sleep–AD bidirectional relationship opens transformative research avenues for clinical intervention. A promising approach involves investigating whether optimized sleep parameters can reduce AD risk or delay progression from preclinical to symptomatic stages. This requires targeted studies in high-risk cohorts—particularly individuals with either preclinical AD biomarkers or autosomal dominant AD mutations—to enable efficient hypothesis testing within practical timelines.

## 5. Current Therapeutic Strategies for Sleep Disorders and AD

### 5.1. Therapeutic Status and Medications for Sleep Disorders

Current therapeutic approaches for sleep disorders are stratified by both clinical severity and disease progression, with varying efficacy ranging across different treatment modalities. First-line management of chronic insomnia typically involves cognitive behavioral therapy for insomnia, which is widely recommended as the initial intervention based on strong clinical evidence [110].

Sedative–hypnotic medications remain a common pharmacological intervention for insomnia despite the known limitations. Chronic administration of these agents may lead to the development of tolerance and paradoxically exacerbate sleep disturbances. Current pharmacotherapeutic approaches for insomnia management primarily include three drug classes: benzodiazepines (BZDs), non-benzodiazepine receptor agonists, and sedating antidepressants. BZDs exert their therapeutic effects through the potentiation of γ-aminobutyric acid (GABA) neurotransmission by allosteric binding to specific sites on GABA_A_ receptors (particularly the α1 subunit-containing BZD-1 sites), thereby enhancing GABAergic inhibition throughout the central nervous system [111]. Zolpidem, a non-benzodiazepine hypnotic agent, has gained widespread clinical use owing to its demonstrated efficacy and favorable safety profile relative to traditional BZDs. In clinical practice, low-dose sedating antidepressants are sometimes employed as alternative pharmacotherapies for insomnia. Evidence from controlled studies indicates that certain tricyclic antidepressants (e.g., mirtazapine, trimipramine, and amitriptyline) and serotonin antagonists and reuptake inhibitors (e.g., trazodone) can significantly improve key sleep parameters. These agents have been shown to increase total sleep time, enhance sleep quality, promote sleepiness, and reduce sleep onset latency [112].

Preclinical and clinical investigations have identified numerous natural products with significant sedative and anxiolytic properties. These botanically derived compounds include honokiol and magnolol from *Magnolia officinalis* bark [113,114], jujuboside A from Semen Ziziphi Spinosae (*Ziziphus spinosa*) [115], sinomenine from *Sinomenium acutum* [116], decursinol from *Angelica gigas* [117], longan aril from *Euphoria longan* [118], ginsenosides from *Panax ginseng* [119], luteolin derivatives in *Chrysanthemum morifolium* [120], and apigenin from *Matricaria chamomile* [121]. Each of these bioactive constituents modulates distinct neuropharmacological pathways associated with sleep regulation [122,123,124,125], but the majority exert their effects through GABAergic mechanisms [115,121]. As GABA_A_ receptor agonists, they primarily bind to neuronal surface receptors, facilitating chloride ion channel opening and subsequent membrane hyperpolarization [126]. The resulting chloride ion influx induces neuronal hyperpolarization, thereby decreasing postsynaptic excitability [127]. This GABA_A_ receptor-mediated inhibition further leads to the dampening of central nervous system arousal, a reduction in sympathetic tone, and the promotion of psychophysiological relaxation [127,128]. Through its inhibitory modulation of neuronal excitability, GABA reduces overall cerebral activity—a fundamental neurophysiological mechanism facilitating both sleep initiation and sleep maintenance processes [129,130,131]. However, GABA-modulating therapies are associated with several adverse effects, most notably daytime sleepiness, irritability, nausea, and cephalalgia. Furthermore, the therapeutic efficacy of natural GABAergic compounds is frequently compromised by person-to-person variability and subsequent bioactive constituents.

### 5.2. Therapeutic Status and Medications for AD

In AD pharmacotherapy, only two drug classes currently have regulatory approval: acetylcholinesterase (AChE) inhibitors and N-methyl-D-aspartate (NMDA) receptor antagonists. AD pathogenesis involves multiple mechanisms that selectively impair cholinergic neurons, leading to widespread deficits in cholinergic neurotransmission. AChE inhibitors—categorized as reversible, irreversible, or pseudo-irreversible—exert their therapeutic effect by inhibiting both AChE and butyrylcholinesterase, thereby preventing ACh catabolism and increasing its synaptic availability [132,133]. Pathological overactivation of NMDA receptors triggers excessive calcium ion (Ca^2+^) influx, leading to excitotoxic neuronal apoptosis and synaptic dysfunction [134]. NMDA receptor antagonists exert neuroprotective effects by attenuating receptor hyperactivation, reducing pathological Ca^2+^ influx, and restoring physiological neuronal signaling. While both AChE inhibitors and NMDA receptor antagonists demonstrate symptomatic efficacy in AD management, it is crucial to note that these pharmacotherapies provide only palliative benefits, neither halting disease progression nor addressing underlying neuropathology [135]. Studies suggest that IL-1β-mediated spatial memory deficits specifically impair ACh release during memory retrieval [136,137], identifying neuroinflammation suppression as a promising therapeutic target for AD intervention [137]. Current potential therapeutic strategies for AD are targeting multiple pathological mechanisms, including the normalization of tau protein phosphorylation and Aβ aggregation, enhanced clearance of Aβ plaques, modulation of neuroinflammatory pathways, and attenuation of oxidative stress through free radical scavenging. These approaches aim to develop disease-modifying treatments that address the underlying neuropathology rather than merely alleviating symptoms [138].

In contrast to non-modifiable risk factors, many AD risk factors related to brain health and lifestyle are amenable to non-pharmacological intervention. Robust evidence indicates that regular physical exercise confers neuroprotective benefits through multiple mechanisms, enhancing cerebral angiogenesis and vascular integrity, promoting synaptic plasticity and hippocampal neurogenesis, and attenuating neuroinflammation by reducing Aβ deposition [139,140]. These exercise-induced adaptations collectively preserve cognitive function in aging populations. Emerging evidence also suggests the Mediterranean diet—particularly rich in n-3 polyunsaturated fatty acids (PUFAs)—combined with sustained cognitive engagement and higher educational attainment appears to enhance neurocognitive function through multiple complementary mechanisms. Comprehensive studies demonstrate that multidomain interventions incorporating dietary modification, regular physical activity, cognitive stimulation, and optimized sleep hygiene can effectively preserve cognitive capacity in aging populations [141]. In this review, we highlight n-3 FAs as a promising therapeutic alternative, given their well-documented multidomain bioactivity, neuroprotective properties, and favorable safety profile.

## 6. Therapeutic Potential of n-3 PUFAs in Sleep Disorders and AD

### 6.1. Dietary Sources of n-3 PUFAs

n-3 PUFAs are predominantly obtained through dietary sources, with marine organisms representing the richest reservoirs. Fatty fish (such as salmon, mackerel, and sardines) and shellfish (including oysters, mussels, and shrimp) contain particularly high concentrations, with wild-caught varieties exhibiting significantly greater n-3 PUFA content than their farmed counterparts due to their natural phytoplankton- and zooplankton-based diets [142]. Notably, cold-water fish species have evolved to accumulate higher proportions of long-chain (LC) n-3 PUFAs as an adaptive response to low-temperature environments. Plant-based sources primarily provide the precursor α-linolenic acid (ALA), which is found in appreciable quantities in flaxseeds, chia seeds, walnuts, and certain oils (flaxseed, canola, and olive), while green leafy vegetables contain modest amounts [143]. In contrast, n-6 PUFAs are abundant in common vegetable oils (corn, soybean, and sunflower) and processed foods. Modern Western diets typically demonstrate an imbalanced n-6:n-3 ratio (15–20:1), which is substantially higher than the recommended 5:1 ratio, largely due to the limited dietary sources of n-3 PUFAs coupled with excessive consumption of n-6-rich products [144]. This nutritional imbalance has significant implications for inflammatory regulation and neurological health.

### 6.2. Importance of Homeostatic Balance Between Omega-3 and Omega-6 PUFAs

**Figure 5:**
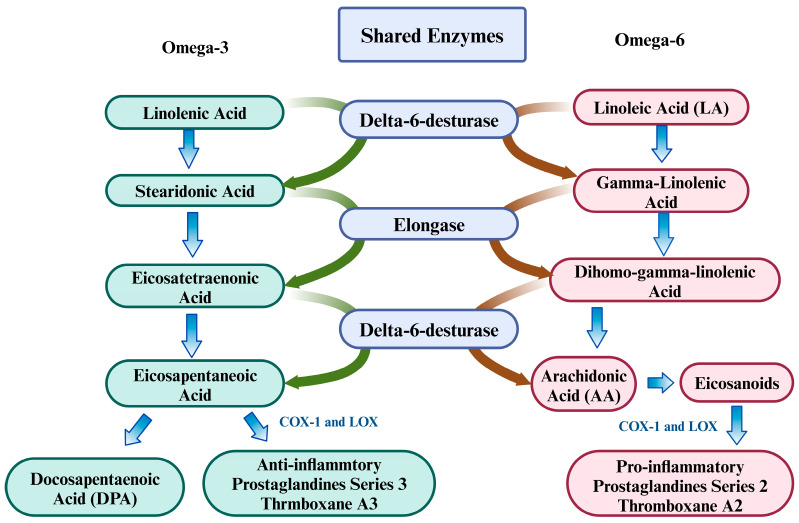
Conversion of n-3 and n-6 into their derivatives using shared enzymes.

n-3 PUFAs possess well-documented nutritional and therapeutic properties, including antioxidant, anti-inflammatory, and neurogenic effects [145,146]. The PUFA family consists of ALA and linoleic acid (LA), which are essential for cellular and neurological function as precursors for LC-PUFAs. Through sequential desaturation and elongation using shared enzymes, LA is converted to arachidonic acid (AA) [147], while ALA yields eicosapentaenoic acid (EPA), docosapentaenoic acid (DPA), and docosahexaenoic acid (DHA) [148]. AA-derived eicosanoids—particularly prostaglandins (PGs) and leukotrienes (LTs)—are potent mediators of inflammation and immune activation [149]. This link is underscored by preclinical and clinical observations, which denote that elevated n-6 PUFA dietary intake correlates with increased proinflammatory markers, including C-reactive protein and IL-6 [150,151]. The cyclooxygenase enzymes (COX)-1 and COX-2 catalyze AA and generate prostaglandin (PG) derivatives, including PGE_2_, PGI_2_, PGD_2_, and PGF_2_α [152]. Series-2-prostaglandins (PGE_2_, PGD_2_, and PGF_2_α) potently stimulate proinflammatory cytokine production, including IL-6. In a parallel pathway, arachidonate 5-lipoxygenase (LOX) converts AA into 5-hydroxyeicosatetraenoic acid and 5-hydroperoxyeicosatetraenoic acid, key precursors for leukotriene biosynthesis [153,154]. These 5-LOX derivatives generate both four-series leukotrienes (LTA_4_, LTC_4_, LTD_4_, LTE_4_, and LTB_4_). As a potent inflammatory mediator, LTB_4_ activates neutrophil recruitment and chemotaxis while stimulating lysosomal enzyme release, ROS generation, and vascular hyperpermeability [155,156]. LTB_4_ also stimulates the production of proinflammatory cytokines, such as IL-6 and IL-1β [157]. The involvement of n-6 PUFAs and their eicosanoid derivatives is implicated in Aβ deposition, a key characteristic of AD onset and progression [158]. A schematic diagram for this conversion is given below (Figure 5).

### 6.3. Therapeutic Potential of Omega-3 Fatty Acids

Unlike AA, EPA and DHA preferentially incorporate into cell membranes, displacing AA and reducing its bioavailability. This competitive inhibition decreases substrate availability for COX-2 and 5-LOX, thereby attenuating their proinflammatory oxidative activity [159]. This inhibitory action also reduces the release of proinflammatory cytokines, e.g., IL-1β, TNF-α, and IL-6. Another key anti-inflammatory mechanism of n-3 PUFAs involves NFκB pathway suppression by modulating classical tools, like the receptor (TLR)-4 downstream activation of inhibitory kappa B [160], which modulate the activation of NFκB. By inhibiting NFκB activation during early inflammatory signaling, EPA/DHA can significantly reduce downstream proinflammatory cytokine production, including TNF-α, IL-1β, and IL-6 [161] by inhibiting the conversion of PGE_2_ and LTs into LT_2_ and LT_4_, which reduces leukocyte-mediated inflammatory responses [162]. Conversely, as inefficient COX/LOX substrates, EPA/DHA reduces inflammatory eicosanoids by reducing PGE_2_ and inhibiting TLR2-driven COX-2 in microglia. Concurrently, it blocks TLR2/4 activation via impaired dimerization/translocation, suppressing immune cell responses and demonstrating layered anti-inflammatory protection [162,163,164,165].

EPA exhibits neuroprotective effects by competitively displacing AA from membrane phospholipids (PLs) and inhibiting COX-mediated oxidation, thereby reducing inflammatory responses and eicosanoids from EPA. Three-series PGs directly suppress the production of AA-derived two-series PGs. Compared to these two-series PGs (e.g., proinflammatory PGE_2_), three-series PGs generally exhibit anti-inflammatory properties [165]. Specifically, PGE_3_, by inhibiting interferon (IFN)-γ, can reduce M1 polarization. Instead, it promotes IL-4-mediated M2 polarization, which can add to the neuroprotective effects of EPA [152,166,167,168].

In addition to their anti-inflammatory effects, n-3 PUFAs within cell membranes are crucial for maintaining appropriate membrane structures and influencing lipid rafts, thereby ensuring the proper environment for membrane protein function [169,170]. Disruptions to this micro-cellular environment can alter gene expressions, protein interactions, and the transportation of molecules across membranes because EPA and DHA are crucial structural elements of membrane PLs in the brain [171,172,173]. Second messengers are produced due to the enzymatic hydrolysis of PLs present in the membranes, e.g., diacylglycerol, and act as substrates for the release of non-esterified PUFAs, which serve as signaling molecules and ligands (or ligand precursors) for transcription factors, such as peroxisome proliferator-activated receptors [174,175]. These substances participate in regulating numerous cellular and tissue responses, as well as the membrane’s physical characteristics. Distinct tissues, cells, and plasma lipid pools exhibit unique FA compositions [176]. For instance, dietary supplementation with fish oil leads to increased levels of DHA and EPA in plasma lipids, leukocytes, erythrocytes, and various tissues/organs, including the brain [177,178]. Epidemiological and clinical research consistently indicates an inverse correlation between decreased dietary/blood plasma concentrations of LC n-3 PUFAs and cognitive performance and a positive correlation with the onset of AD [179]. In several animal models, reduced brain DHA levels resulting from dietary n-3 PUFA deficiency have been linked to memory impairment, compromised neuronal plasticity, and neuroinflammation [180]. Collectively, these studies indicate the therapeutic potential of n-3 PUFAs and highlight their role in the homeostatic functioning of neuroimmunological and neurochemical processes in the brain.

### 6.4. Omega-3 as a Possible Therapeutic Agent for Treating the Pathogenesis of AD

**Figure 6:**
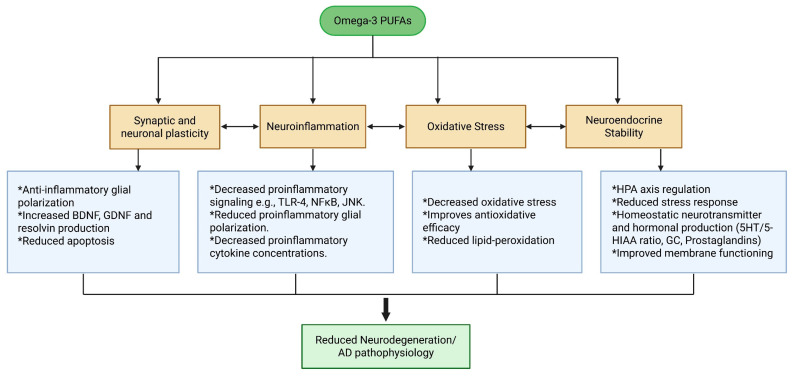
Therapeutic effects of n-3 on pathophysiological mechanisms and progression of AD.

Current AD treatments fail to address the full spectrum of AD-related pathological changes, but n-3 PUFAs, due to their diverse biological activities, can play a multifaceted role in mitigating AD-related pathophysiological changes, as shown in Figure 1 and Figure 6.

LC n-3 PUFAs can modulate neurotransmission, brain development, neuroplasticity, and cellular signaling [12]. The experimental and epidemiological evidence indicates that n-3 FAs may serve as a potential protective factor against early-stage or mild AD. Several studies have demonstrated an association between low dietary intake of n-3 PUFAs and an elevated risk of cognitive decline, dementia, and AD. A positive correlation between dietary or plasma EPA/DHA levels and memory function in healthy adults has been reported [181]. Conversely, studies show that individuals with mild cognitive impairments (MCI) and AD, frequently exhibit lower levels of EPA and DHA in their plasma [182,183]. Moreover, AD patients’ brain samples indicated lower DHA concentrations compared to those of cognitively normal individuals [184]. A prior study found that higher plasma DHA levels were associated with a 47% reduced risk of all-cause dementia and a 39% lower risk of developing AD [185]. Furthermore, long-term supplementation (6 months) with EPA and DHA has demonstrated the potential to alleviate depressive symptoms and improve cognitive functions in adults experiencing MCI [186]. Some of the therapeutical aspects of AD-related pathological changes are detailed below.

#### 6.4.1. The Effects of Omega-3 on Oxidative Stress

Oxidative stress interacts with key AD pathologies, including Aβ plaque formation, tau hyperphosphorylation, and synaptic dysfunction, creating a vicious cycle of neuronal injury and neurodegeneration [90]. n-3 FAs, particularly EPA and DHA, are known for their antioxidative properties. As integral components of brain cell membranes, they play crucial roles in maintaining membrane structural integrity and facilitating molecular transport (Aβ in the context of AD) across the cellular barrier. Their unique biophysical properties allow for the direct modulation of membrane-associated enzymatic activity, particularly through interactions with lipid raft domains. Studies suggest that AA is more prone to lipid peroxidation than n-3 [187], and, as mentioned earlier, EPA and DHA can replace AA from cellular membranes [159]. A detailed meta-analysis indicated that n-3 intake increased the total antioxidative capacity and glutathione peroxidase and decreased malondialdehyde levels [187]. Phospholipase A_2_ (PLA_2_) serves as the rate-limiting enzyme in the AA-derived eicosanoid PGE_2_ cascade. Research demonstrates that Aβ triggers the neuroinflammatory activation of glial cells and induces neuronal apoptosis in AD through oxidative signaling pathways mediated by both cytosolic (c)PLA_2_ and secretory (s)PLA_2_ isoforms [188]. Furthermore, reduced DHA levels in AD may be linked to elevated ROS, which contributes to membrane DHA depletion, ultimately fostering cellular damage and cognitive dysfunction. In Aβ-treated SH-SY5Y neuroblastoma cells, EPA significantly reduced NO and ROS levels. This antioxidant effect appears to be mediated through EPA’s inhibitory action on both neuronal NADPH oxidase and COX-2 enzymatic activity [189]. Furthermore, n-3 can regulate the nuclear factor erythroid 2-related factor (Nrf)2, cAMP response element-binding protein (CREB), and protein phosphatase (PP)2A pathways, which are important for the antioxidative potential of the brain, and any disruption can lead to neuronal damage, synaptic plasticity, and apoptosis, resulting in neurodegeneration. DHA and EPA can facilitate the release of Nrf2 from its inhibitor Keap1 and assist Nrf2 migration into the nucleus, where it binds to the antioxidant response element. n-3 can also significantly elevate the BDNF/TrkB/CREB pathway and restore antioxidant potential by upregulating Nrf2/HO-1 [190,191].

#### 6.4.2. The Effects of Omega-3 on Neuroinflammation and Aβ Clearance

As mentioned earlier, n-3 PUFAs can modulate neuroinflammation by decreasing the conversion of n-6 into inflammation-inducing eicosanoids and leukotrienes, and neuroinflammation is a major contributor to Aβ deposition. Glial polarization is an important aspect of neuroinflammation, and studies have indicated that n-3 can ameliorate the microglial transition into a proinflammatory state, leading to reduced proinflammatory cytokine production [192,193]. Another study indicated that n-3 reduced inflammatory microglial M1 polarization and restored LRP-1 functioning [194]. Additionally, n-3 also recovered mitochondrial impairment induced by Aβ plaques [195,196]. In another study, EPA mitigated interleukin-1β (IL-1β)-induced dopaminergic impairment in the nucleus accumbens. This neuroprotective effect was mechanistically linked to the downregulation of cPLA_2_ expression and subsequent reduction in PGE_2_ biosynthesis [197]. APP and all APP secretases are transmembrane proteins that process APP at sites proximal to and within the lipid bilayer [198].

This highlights that dietary supplementation with n-3 PUFAs may offer therapeutic potential for early-stage and mild AD, as Aβ peptides are generated through the sequential proteolytic cleavage of APP, as mentioned above. Prior research has demonstrated that COS-7 cells transfected with pCEP-SP-C99, DPA, DHA, and docosatrienoic acid Aβ_42_ concentrations were significantly reduced, and Aβ plaque formations were reduced [199]. n-3 reduces the chances of neurotoxic Aβ production and plaque formation by altering APP processing and Aβ clearance from the brain. Studies also suggest that n-3 PUFA-rich shrimp oil counteracted Aβ-induced changes and improved overall cell viability, oxidative stress (ROS/GSH), neurotrophic (NGF/TrkA), inflammatory (TNF-α), and apoptotic (Bax/Bcl-2, Caspase-3) markers in neuroblastoma cells (SH-SY5Y) [74].

#### 6.4.3. The Effects of Omega-3 on Tau Hyperphosphorylation

n-3 PUFAs can modulate tau hyperphosphorylation by employing different mechanisms. Studies have reported that quinolinic acid (QA) contributes to tau hyperphosphorylation, thereby inducing both neuronal dysfunction and synaptic impairment [200]. QA, which is exclusively produced by brain microglia, acts as an NMDA receptor agonist that potentiates excitotoxic neuronal damage. Elevated levels of both IFN-γ and IDO have been documented in the cerebral cortex of female triple-transgenic (3×Tg)–AD mice, which represent a well-established preclinical model of AD pathogenesis [201]. Enhanced tryptophan catabolism through upregulated IDO activity reduces cerebral tryptophan bioavailability, consequently diminishing serotonin and melatonin synthesis. This metabolic shift simultaneously elevates the production of neurotoxic kynurenine pathway metabolites, particularly QA and 3-hydroxykynurenine (3-HK). While 3-HK exacerbates oxidative stress, QA is linked with tau hyperphosphorylation, promoting neurodegeneration and accelerating AD progression, as mentioned earlier [202].

JNK activation and associated phosphorylation are another important aspect of tau hyperphosphorylation and DHA intake that can significantly reduce the hyperphosphorylation and fragmentation of tau due to their suppressive effects on this activation [203]. Endoplasmic reticulum stress likely contributes to insulin signaling impairment in AD by promoting JNK-dependent serine phosphorylation of insulin receptor substrate-1. Phosphorylation of this protein has been further implicated in the pathological processes of Aβ deposition into senile plaques and tau hyperphosphorylation into NFTs [204,205]. However, another study indicated that DHA can reduce the hyperphosphorylation of tau by ameliorating GSK-3β activity [206,207].

More importantly, studies have also attributed an overall higher intake of n-3 with a lesser increase in pTau181 levels (*β* = −0.037 log-transformed pg/mL, *p* = 0.001, *p*-trend = 0.006). This correlation was also examined for EPA and DHA individually (EPA, *β* = −0.033, *p* = 0.004, *p*-trend = 0.016; DHA, *β* = −0.030, *p* = 0.009, *p*-trend = 0.027) to signify the effects of different subtypes of n-3 PUFAs. This significantly supports the notion that n-3 intake can significantly slow down the progression of AD [208].

#### 6.4.4. Other Therapeutic Effects of Omega-3 on Pathophysiology of AD

Another potential mechanism involves n-3 PUFAs directly influencing AD’s neuropathological markers. ApoE-4 is widely recognized as the primary genetic risk factor for AD. As the predominant lipoprotein in the brain, ApoE performs essential functions in cerebral lipid metabolism, which consists of neural membranes and is critically involved in Aβ clearance from the brain. Moreover, multiple lipidomic studies have demonstrated reduced low-density lipoprotein concentrations in both postmortem CSF and brain tissue of AD patients. Conversely, elevated levels of very high-density lipoprotein appear to exacerbate AD pathogenesis [209,210,211]. As previously noted, membrane properties, including fluidity and bilayer thickness—which critically influence intra- and intercellular signaling—are modulated by PL composition. This is particularly relevant given the well-documented reduced DHA levels observed in AD brains [212]. Reductions in DHA levels can significantly modulate multiple neurobiological processes important to AD pathogenesis, including neurotransmission efficiency, ion channel kinetics, the activity of membrane-bound enzymes, transcriptional regulation, glial immune responses, and synaptic plasticity mechanisms [213]. Another key aspect of AD is cholinergic deficiency, and this is a target of current AD-related therapies. In this context, n-3 PUFAs can act as a precursor for protectin D1, which helps elevate serotonin and ACh levels in neural tissues [214].

In conjunction, n-3 can ameliorate or reduce the progression of AD by modulating multiple neuropathological factors. Some clinical studies that validate the therapeutic potential of n-3 in AD are given in Table 1.

It is important to note that the negative results in DeVore’s studies can be attributed to the cooking method and type of fish, as the authors noted that the participants used lean fish instead of fatty fish, which is full of n-3 FAs [221]. This signifies the importance of the type of fish and cooking method for proper n-3 PUFA supplementation.

### 6.5. The Possible Therapeutic Roles of Omega-3 for Treating Sleep Disorders

Recent investigations into the effects of LC n-3 PUFAs on sleep regulation have intensified. Animal studies demonstrate that n-3 LC-PUFAs may influence sleep physiology through key mechanisms, i.e., the modulation of melatonin biosynthesis, reducing neuroinflammatory pressure, maintaining the brain structure/function, and the maintenance of neuronal membrane integrity and circadian regulation [223,224,225,226,227,228], as mentioned earlier. n-3 can act as a non-photic zeitgeber and circadian synchronizer [228] and can possibly ameliorate HPA axis hyperactivation, both of which are important for sleep induction and better sleep quality, by facilitating the formation of a corticosterone-basic helix–loop–helix ARNT-like protein-1 complex. This complex activates a negative feedback loop that regulates HPA axis activity at the promoter region of the period circadian protein, which contains the glucocorticoid response element–E-box region, where this complex is formed [229,230,231]. n-3 FAs can improve tryptophan concentration in the brain by increasing the BBB fluidity, improving tryptophan metabolism, and reducing the competition for tryptophan transportation across the BBB by decreasing the ratio between free tryptophan and branched-chain amino acids [19,232,233,234]. These changes increase the bioavailability of tryptophan in the brain, which is the basic raw material for serotonin and, ultimately, melatonin. The inhibition of neuroinflammatory cascades also reduces the catabolic activity of IDO, which further enhances the production of serotonin and melatonin. Another mechanism that n-3 employs to improve sleep onset and latency is its effects on the oligomerization kinetics of glycoprotein-coupled receptors, such as adenosine A_2A_ and dopamine D2 receptors. Both of these receptors facilitate neuron excitability, and DHA can modulate the oligomerization circle of these receptors and decrease neuronal excitability and, in conjunction with other studies, can improve sleep latency [234,235].

These processes are essential for both sleep onset and sleep maintenance. Clinical studies in humans have corroborated these findings, demonstrating that both n-3 LC-PUFA supplementation and dietary intake (particularly through fatty fish consumption) significantly improve multiple sleep parameters, such as reduced sleep latency, increased total sleep duration (particularly during recovery sleep periods), and enhanced overall sleep quality [236,237,238,239]. Clinical studies demonstrate that n-3 PUFA deficiency exacerbates sleep disturbances and reduces sleep quality across both pediatric and adult populations [238]. Conversely, adequate dietary intake of n-3 PUFAs—particularly EPA and DHA—shows significant efficacy in improving sleep quality metrics [240]. Table 2 summarizes key clinical studies examining the role of n-3 FAs in sleep regulation.

While the existing evidence suggests that n-3 LC-PUFAs may offer potential benefits as a dietary supplement for improving sleep parameters in both pediatric and adult populations, the current research landscape reveals notable inconsistencies. Several well-controlled clinical trials have failed to demonstrate significant improvements in sleep quality following n-3 LC-PUFA supplementation, particularly in specific clinical populations, including patients with chronic insomnia disorder and peri- and postmenopausal women with sleep disturbances [249]. The observed heterogeneity in research outcomes may be attributable to several methodological factors, including variations in study design, participant characteristics, and sleep assessment methodologies. Consequently, while the preliminary evidence suggests potential benefits, the therapeutic efficacy of n-3 LC-PUFA supplementation for sleep improvement requires more rigorous investigation.

## 7. Conclusions, Limitations, and Future Directions

The complex interplay between sleep disturbances and AD necessitates a deeper exploration beyond mere correlation. This review introduces sleep disorders—especially SD—as a potential precursor/initiator to AD, supported by evidence highlighting its detrimental impact on neuropathological cascades linked to AD pathogenesis. Given the variability in response to current SD and AD therapeutic interventions, n-3 PUFAs emerge as a promising therapeutic option due to their wide range of neurophysiological and bioactive properties. However, challenges such as food–food interactions and bioavailability remain significant barriers [250]. By utilizing transgenic models like Fat-1 mice, which endogenously synthesize n-3 FAs, such limitations can be addressed. Some other harmful effects induced by n-3 FAs include contamination with mercury and organic pollutants, which can affect vulnerable populations, particularly breastfeeding women [251].

Future research should prioritize mechanistic studies to unravel the sleep–AD nexus, targeted interventions for high-risk groups, and circadian-based therapies to optimize sleep patterns. In the near term, sleep optimization trials among presymptomatic AD mutation carriers and biomarker-positive elders, combined with advances in wearable sleep monitoring and fluid-based AD detection, offer promising translational pathways.

## Figures and Tables

**Table 1:** Therapeutic effects of n-3 on AD in different clinical settings.

No.	Study	Subjects	Study Type	Treatment/Source	Duration	Results
1	Freund-Levi et al. [215]	174 AD patients	Randomized double blind trial	DHA: 1.7 g; EPA: 0.6 g	6 months	n-3 intake improved AD-induced cognitive decline in very mild cases
2	Liu et al. [216]	215,083 healthy adults (≥60 y)	Prospective cohort study	Intake of fish oil supplements	7.92 years	A marginal interaction between ApoE gene variants, n-3, and risk of cognitive decline was found
3	Lin et al. [217]	163 MCI or AD patients	Randomized placebo-controlled trial	DHA: 0.7 g; EPA: 1.6 g; EPA + DHA: 0.8 + 0.35 g	2 years	EPA reduced CCL4 levels and the constructional praxis. Both EPA and DHA improved the speaking ability score
4	Shinto et al. [218]	39 AD patients	Randomized placebo-controlled trial	DHA: 675 mg; EPA: 975 mg; LA: 600 mg	1 year	The combination reduced the rate of decline in MMSE scores in AD patients
5	Gustafon et al. [219]	2612 healthy elderly (≥65 y)	Prospective cohort study	n-3 PUFA dietary supplement	7 years	n-3 intake is positively correlated with lowered risk of developing AD
6	Gu et al. [220]	1219 healthy elderly (≥65 y)	Prospective cohort study	Mediterranean diet rich in n-3 intake	4 years	Mediterranean diet intake is associated with reduced risks of AD
7	DeVore et al. [221]	5395 healthy adults (≥55 y)	Population-based cohort study	n-3 and fish intake	9.6 years	No significant changes in memory were found between the treatment and placebo groups
8	Danthiir et al. [222]	403 healthy elderly (65–90 y)	Double-blind, randomized, placebo-controlled	EPA: 600 mg; DHA: 1720 mg	18 months	n-3 intake showed a positive correlation in ApoE carriers

**Table 2:** Therapeutic effects of n-3 on sleep in different clinical settings.

No.	Study	Subjects	Study Type	Treatment/Source	Duration	Results
1	Yokoi-Shimizu et al. [241]	66 healthy Japanese males and females	Double-blinded, placebo-controlled trial	DHA: 576 mg; EPA: 284 mg	12 weeks	Sleep quality improved compared to placebo
2	Murphy, Rachel A. et al. [242]	6175 adult participants with insomnia	Observational cross-sectional study	Serum fatty acid analysis	1 year	Poor sleep quality was associated with reduced blood EPA and DHA levels
3	Yehuda et al. [243]	126 students suffering from anxiety	Randomized controlled trial	Fatty acid mixture treatment	One month	Fatty acid mixture improved sleep quality
4	Dretsch et al. [244]	160 US male and female soldiers (18–55 y)	Randomized, double-blind, placebo-controlled trial	2.5 g of EPA + DHA ethyl esters/day	60 days	Decreased daytime sleepiness
5	Ford PA et al., [245]	8771 healthy participants	Observational longitudinal study	n-3 PUFA dietary supplement	4 years	n-3 intake is positively correlated with sleep duration
6	Christian LM et al. [246]	135 pregnant females	Observational longitudinal study	RBC DHA contents	2nd trimester till partum	RBC DHA contents were positively correlated with sleep quality and duration
7	Lotrich FE et al. [247]	104 non-depressed hepatitis C patients	Prospective observational study	AA/EPA + DHA status	6 months	Serum n-3 status is positively correlated with sleep quality and reduced MDD
8	Liu J. et al. [248]	581 schoolchildren (9–11 y)	Prospective cohort study	Fish consumption	2 years	Fish intake decreased the sleep disturbance score
9	Patan. et al. [240]	84 young healthy adults (29–45 y)	Randomized controlled trial	DHA: 900 mg EPA: 270 mg	26 weeks	EPA and DHA intake improved sleep latency and sleep efficiency

## Data Availability

Data sharing is not applicable to this article.

## Abbreviations

The following are the commonly used abbreviations in this manuscript:

AD	Alzheimer’s disease
SD	Sleep deprivation
Aβ	Amyloid-beta
LC n-3 PUFAs	Long-chain omega-3 polyunsaturated fatty acids
IL	Interleukin
PLs	Phospholipids
SWA	Slow-wave activity
APP	Amyloid precursor protein
ROS	Reactive oxygen species
LRP-1	Low-density lipoprotein receptor-related protein 1
BACE-1	Beta-secretase 1
RAGE	Receptor for advanced glycation end products
TNFR	Tumor necrosis factor receptor
NMDA	N-methyl-D-aspartate
ApoE	Apolipoprotein E
NFTs	Neurofibrillary tangles
TREM	Triggering receptor expressed on myeloid cells
